# Supplementary material for: Vinorelbine enhances the efficacy of oncolytic vaccinia virus in a preclinical model of ovarian high-grade serous carcinoma
Source: Mol Ther Oncol. 2025 Dec 3;34(1):201105. doi: 10.1016/j.omton.2025.201105 (PMC13006439; doi:10.1016/j.omton.2025.201105)
Supplement: Document S1. Figures S1–S6 [file mmc1.pdf]

OMTON, Volume *34*

Supplemental information

Vinorelbine enhances the efficacy of oncolytic vaccinia virus in a preclinical model of ovarian high-grade serous carcinoma

Stephanie Drymiotou, Christophe J. Queval, Katherine E. Tyson, Lesley A. Sheach, Antonio Postigo, Ilaria Dalla Rosa, Darren P. Ennis, Michael Howell, Iain A. McNeish, and Michael Way

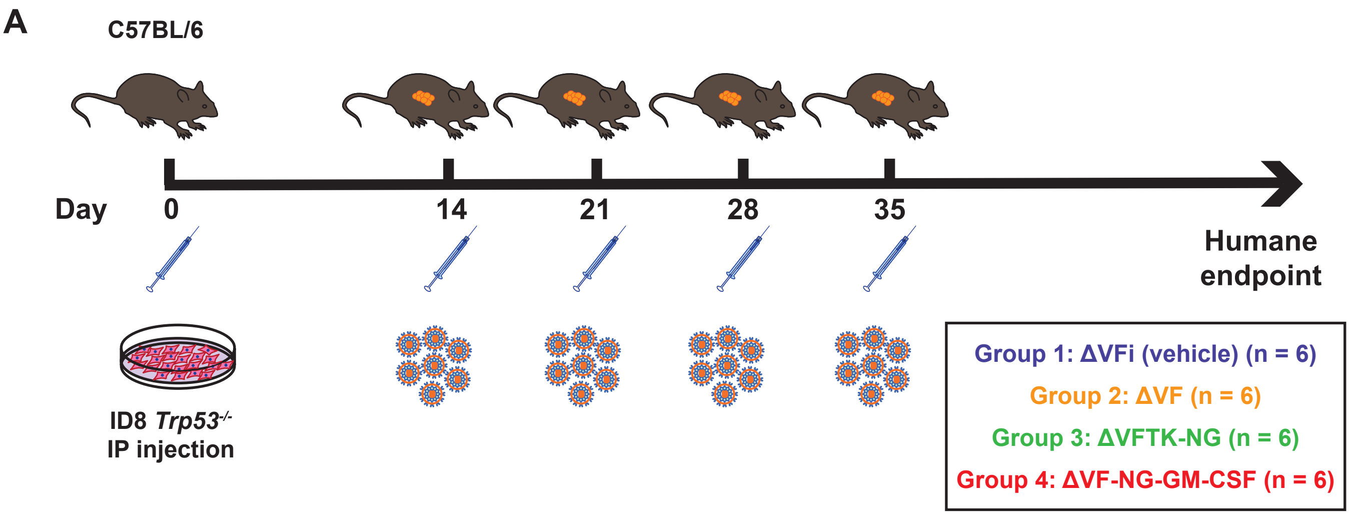

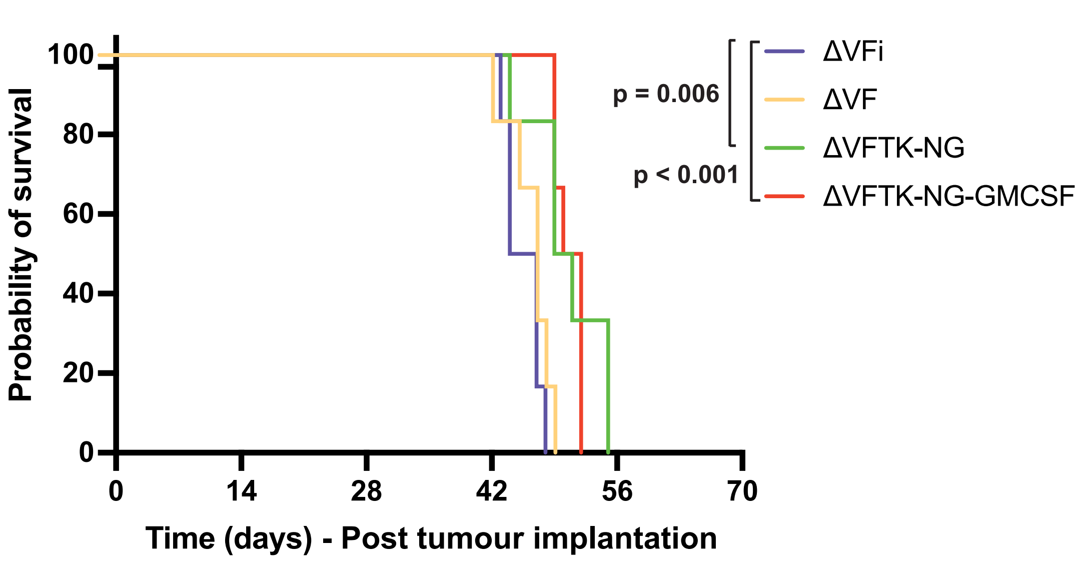

C

	ΔVFi	ΔVF	ΔVFTK-NG	ΔVFTK-NG-GM-CSF
**Omental tumour (mg)**	112.0 ± 13.0	93.0 ± 8.0	100.0 ± 3.8	89.0 ± 6.6
**Spleen (mg)**	365.0 ± 31.0	274.0 ± 31.0	306.0 ± 15.0	310.0 ± 40.0
**Liver (mg)**	848.0 ± 61.0	839.0 ± 39.0	759.0 ± 45.0	808.0 ± 33.0
**Ascites (ml)**	4.7 ± 0.5	3.0 ± 0.5	5.4 ± 0.7	5.6 ± 0.8

Figure S1. ΔVFTK-NG-GM-CSF provides superior survival benefit in monotherapy **A**. Schematic representation of the experimental design of the *in vivo* survival study. Four groups of mice were injected IP with ID8 Trp53$^{-/-}$ cells on day 0 and subsequently inoculated with the indicated viruses on day 14, 21, 28 and 35. ΔVFi is the control heat inactivated virus. **B**. Kaplan-Meier survival curve showing survival data for each virus analysed by log-rank test. **C**. Quantification of omental tumour, spleen and liver weights as well as ascitic volumes for each group. Data are represented as mean ± SD. One-way ANOVA was used to determine significance between all groups with Tukey multiple comparisons post-hoc test.

	1	2	3	4	5	6	7	8	9	10	11	12	13	14	15	16	17	18	19	20	21	22	23	24
A	DMSO	DMSO	AG-1478	Ellipticine (hydrochloride)	TRAM-34	Nolatrexed dihydrochloride	Sitagliptin	MMAF-Ome	Evacetrapib	Erastin	Ceftazidime	MMAD	Enzastaurin	Cytochalasin E	TPCA-1	Aminopterin	IMD-0354	DMSO	Sepantronium (bromide)	DMSO	Olaparib	DMSO	DMSO	DMSO
B	DMSO	DMSO	DMSO	DMSO	DMSO	DMSO	DMSO	DMSO	DMSO	DMSO	DMSO	DMSO	DMSO	DMSO	DMSO	DMSO	DMSO	DMSO	DMSO	DMSO	DMSO	DMSO	DMSO	DMSO
C	DMSO	DMSO	Gemcitabine	Geldanamycin	Iniparib	Idasanutlin	Finasteride	SNX-2112	Chromomycin A3	MMAF (hydrochloride)	CCT 137690	DMSO	Methotrexate	DMSO	Alvespimycin (hydrochloride)	Pemetrexed (disodium)	Cabozantinib	DMSO	Pralatrexate	DMSO	Sumatriptan (succinate)	DMSO	DMSO	DMSO
D	DMSO	DMSO	DMSO	DMSO	DMSO	DMSO	DMSO	DMSO	DMSO	DMSO	DMSO	DMSO	DMSO	DMSO	DMSO	DMSO	DMSO	DMSO	DMSO	DMSO	DMSO	DMSO	DMSO	DMSO
E	DMSO	DMSO	Bortezomib	Harringtonine	Mitoxantrone	AMG131	GW842166X	Imidazole ketone erastin	PF-573228	Thapsigargin	NSC319726	DMSO	Raltitrexed	DMSO	Omipalisib	Dicloxacillin (Sodium hydrate)	CHIR-124	DMSO	Vinorelbine (ditartrate)	DMSO	Erlotinib	DMSO	DMSO	DMSO
F	DMSO	DMSO	DMSO	DMSO	DMSO	DMSO	DMSO	DMSO	DMSO	DMSO	DMSO	DMSO	DMSO	DMSO	DMSO	DMSO	DMSO	DMSO	DMSO	DMSO	DMSO	DMSO	DMSO	DMSO
G	DMSO	DMSO	Clotrimazole	Trifluridine	Tolnaftate	Combretastatin A4	Asimadoline (hydrochloride)	Auristatin F	JNJ-42165279	PU-H71	Flucloxacillin sodium	DMSO	Onalespib	DMSO	Vorinostat	DMSO	GSK2334470	DMSO	TUG-469	DMSO	CCT241736	DMSO	DMSO	DMSO
H	DMSO	DMSO	DMSO	DMSO	DMSO	DMSO	DMSO	DMSO	DMSO	DMSO	DMSO	DMSO	DMSO	DMSO	DMSO	DMSO	DMSO	DMSO	DMSO	DMSO	DMSO	DMSO	DMSO	DMSO
I	DMSO	DMSO	Ruboxistaurin	NVP-2	Dutasteride	Prexasertib	Daunorubicin (hydrochloride)	Rocaglamide	LY2922470	WR99210	Amsacrine (hydrochloride)	Ergosterol	Rosiglitazone	DMSO	AT7519	DMSO	Canertinib (dihydrochloride)	DMSO	Fasiglifam	DMSO	Nutlin-3a	DMSO	DMSO	DMSO
J	DMSO	DMSO	DMSO	DMSO	DMSO	DMSO	DMSO	DMSO	DMSO	DMSO	DMSO	DMSO	DMSO	DMSO	DMSO	DMSO	DMSO	DMSO	DMSO	DMSO	DMSO	DMSO	DMSO	DMSO
K	DMSO	DMSO	Tanespimycin	BRL 54443	Ganetespib	Podofilox	GSK3787	Abexinostat	SNS-032	Carfilzomib	Elesclomol	ZSTK474	MA-0204	DMSO	BX795	DMSO	Tubulin inhibitor 1	DMSO	Torcetrapib	DMSO	JNJ-10198409	DMSO	DMSO	DMSO
L	DMSO	DMSO	DMSO	DMSO	DMSO	DMSO	DMSO	DMSO	DMSO	DMSO	DMSO	DMSO	DMSO	DMSO	DMSO	DMSO	DMSO	DMSO	DMSO	DMSO	DMSO	DMSO	DMSO	DMSO
M	DMSO	DMSO	Roflumilast	Diacetoxyscirpenol	Cytarabine	NVP-HSP990	Trimebutine (maleate)	Silvestrol	Apremilast	Nonactin	CP-91149	FL118	Ceftiofur	DMSO	Floxuridine	DMSO	(R)-CR8	DMSO	NVP-TAE 226	DMSO	Alogliptin	DMSO	DMSO	DMSO
N	DMSO	DMSO	DMSO	DMSO	DMSO	DMSO	DMSO	DMSO	DMSO	DMSO	DMSO	DMSO	DMSO	DMSO	DMSO	DMSO	DMSO	DMSO	DMSO	DMSO	DMSO	DMSO	DMSO	DMSO
O	DMSO	DMSO	PF-3845	CP-316819	Bay 59-3074	Diosgenin	Vindesine (sulfate)	Aleglitazar	Seralutinib	SPDB-DM4	Pazopanib (Hydrochloride)	CH5138303	5-Fluorouridine	DMSO	Vinblastine (sulfate)	DMSO	PF-04691502	DMSO	PS-1145	DMSO	Dinaciclib	DMSO	DMSO	DMSO
P	DMSO	DMSO	DMSO	DMSO	DMSO	DMSO	DMSO	DMSO	DMSO	DMSO	DMSO	DMSO	DMSO	DMSO	DMSO	DMSO	DMSO	DMSO	DMSO	DMSO	DMSO	DMSO	DMSO	DMSO

Solvant Water
Solvant Ethanol
Solvant DMSO

Red: 2 mM concentration
Black: 10 mM concentration

Figure S2. Secondary screen mother plate layout

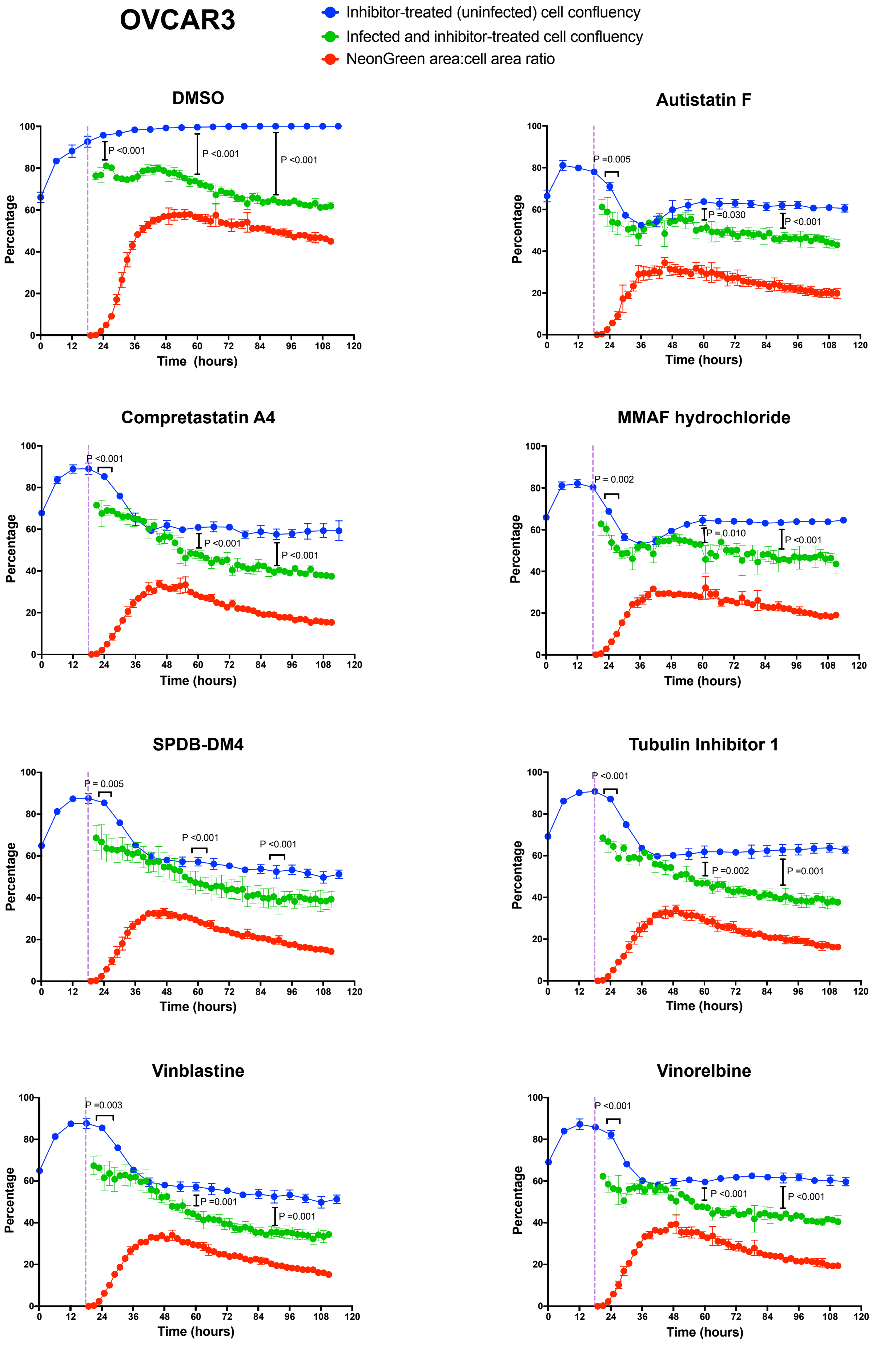

Figure S3. Tubulin assembly inhibitors enhance vaccinia-induced OVCAR3 cell death Representative graphs of the combination of the indicated tubulin polymerisation inhibitors with vaccinia in OVCAR3 cell line. The purple dotted line represents infection with ΔVFTK-NG (MOI 0.5) at 18 hours post cell seeding. All compounds were used at 1 μM. Student's t-test was used to compare uninfected against infected cell confluency at 25, 60 and 90 hours post cell seeding. Error bars represent SD.

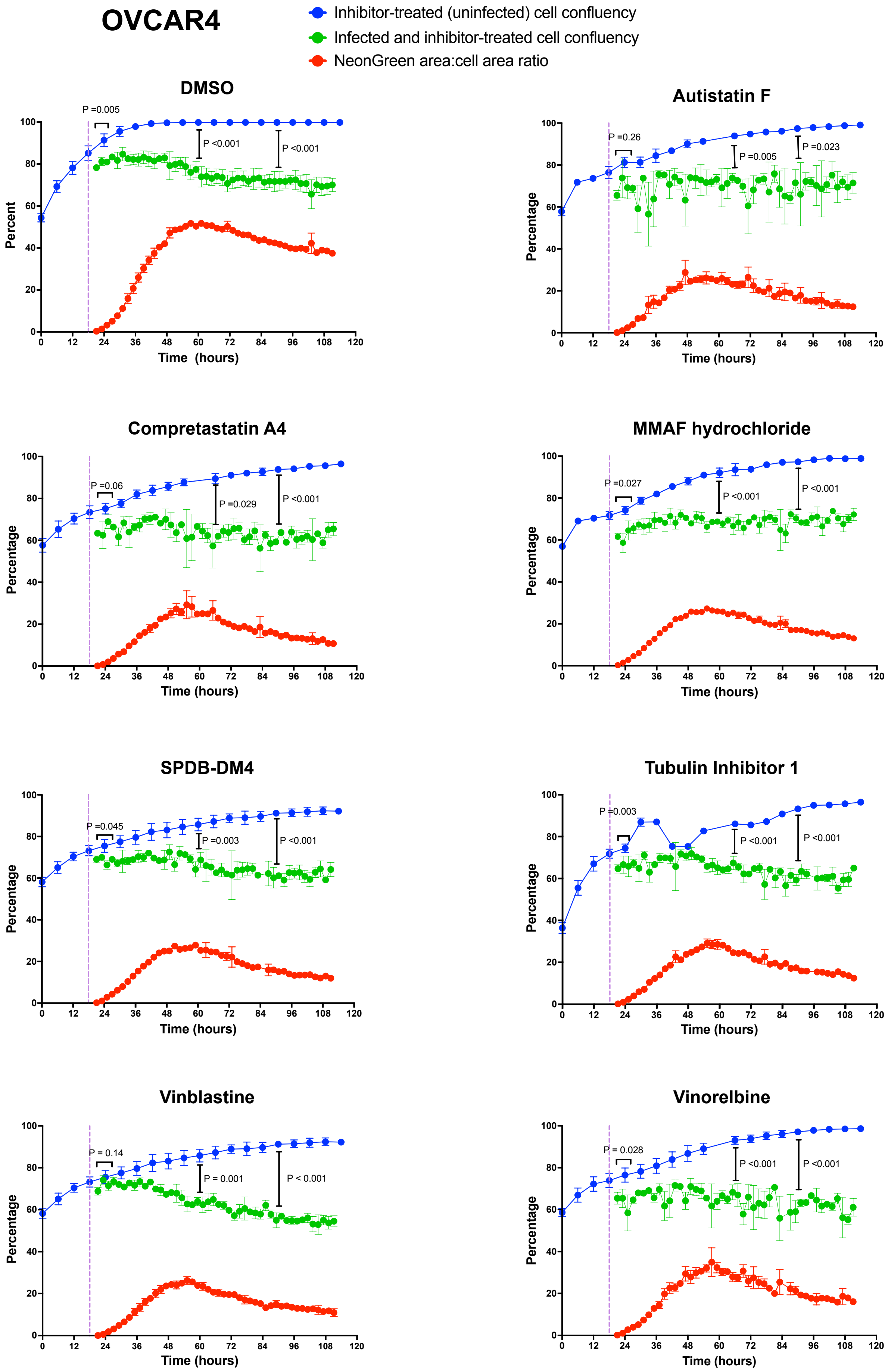

Figure S4. Tubulin assembly inhibitors enhance vaccinia-induced OVCAR4 cell death Representative graphs of the combination of the indicated tubulin polymerisation inhibitors with vaccinia in OVCAR4 cell line. The purple dotted line represents infection with ΔVFTK-NG (MOI 0.5) at 18 hours post cell seeding. All compounds were used at 1 μM. Student's t-test was used to compare uninfected against infected cell confluency at 25, 60 and 90 hours post cell seeding. Error bars represent SD.

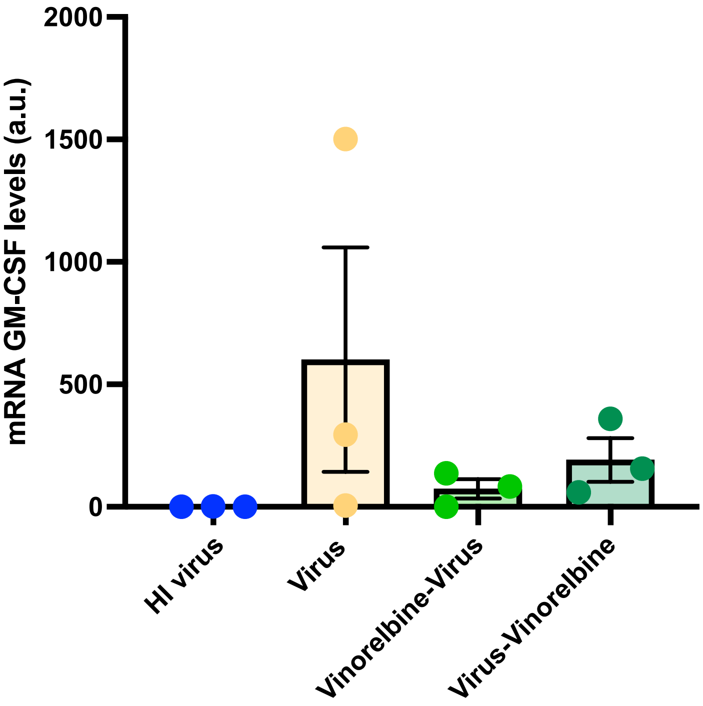

Figure S5. Quantification of GM-CSF mRNA in ΔVFTK-NG-GM-CSF infected tumours Quantification of GM-CSF mRNA in omental tumours infected with virus (ΔVFTK-NG-GM-CSF) with or without vinorelbine treatment. HI represents the heat inactivated control virus control. The tumour samples are from the same experiments shown in Figure 5A. Error bars represent mean ± SD.

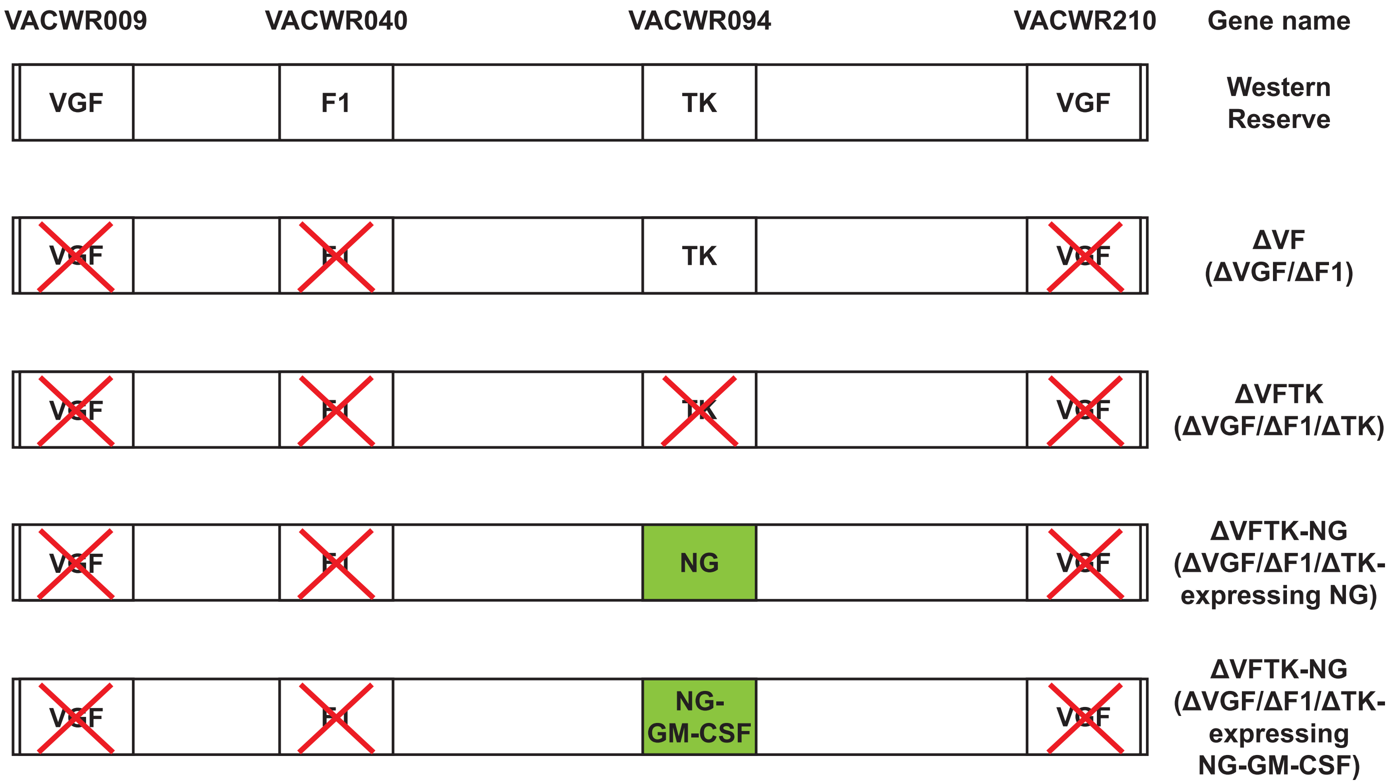

Figure S6. Generation of recombinant viruses
Schematic illustrating the gene modifications made for the generation if the indicated recombinant viruses
